# One-pot synthesis of Ag–In–Ga–S nanocrystals embedded in a Ga_2_O_3_ matrix and enhancement of band-edge emission by Na^+^ doping†

**DOI:** 10.1039/d3na00755c

**Published:** 2023-11-14

**Authors:** Makoto Tozawa, Chie Miyamae, Kazutaka Akiyoshi, Tatsuya Kameyama, Takahisa Yamamoto, Genichi Motomura, Yoshihide Fujisaki, Taro Uematsu, Susumu Kuwabata, Tsukasa Torimoto

**Affiliations:** a Graduate School of Engineering, Nagoya University Chikusa-ku Nagoya 464-8603 Japan torimoto@chembio.nagoya-u.ac.jp; b Science & Technology Research Laboratories, Japan Broadcasting Corporation (NHK) 1-10-11 Kinuta, Setagaya-ku Tokyo 157-8510 Japan; c Department of Applied Chemistry, Graduate School of Engineering, Osaka University Suita Osaka 565-0871 Japan; d Innovative Catalysis Science Division, Institute for Open and Transdisciplinary Research Initiatives (ICS-OTRI), Osaka University Suita Osaka 565-0871 Japan

## Abstract

I–III–VI-based semiconductor quantum dots (QDs) have been intensively explored because of their unique controllable optoelectronic properties. Here we report one-pot synthesis of Na-doped Ag–In–Ga–S (AIGS) QDs incorporated in a Ga_2_O_3_ matrix. The obtained QDs showed a sharp band-edge photoluminescence peak at 557 nm without a broad-defect site emission. The PL quantum yield (QY) of such QDs was 58%, being much higher than that of AIGS QDs without Na^+^ doping, 29%. The obtained Na-doped AIGS/Ga_2_O_3_ composite particles were used as an emitting layer of green QD light-emitted diodes. A sharp electroluminescence (EL) peak was observed at 563 nm, being similar to that in the PL spectrum of the QDs used. The external quantum efficiency of the device was 0.6%.

## Abstract 2

Ag–In–Ga–S nanocrystals embedded in a Ga_2_O_3_ matrix produced a narrow band-edge photoluminescence, the intensity being significantly enlarged by Na^+^ doping, and they effectively served as an emitting layer in green quantum dot-light-emitting diodes.

## Graphical abstract

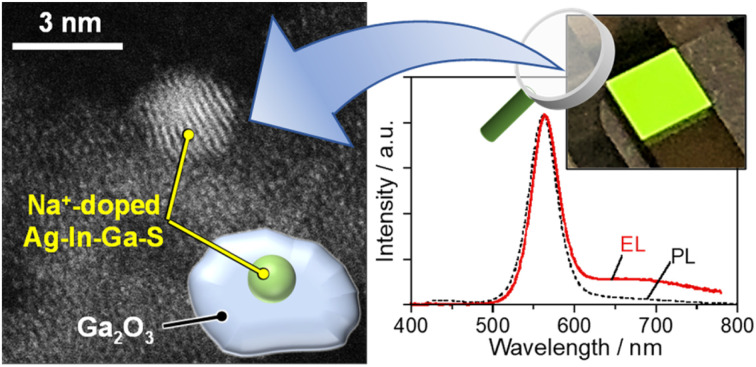

## Introduction

Multinary quantum dots (QDs) have attracted increasing attention for applications to practical devices such as displays,^1–6^ solar cells,^7–10^ photocatalysts,^11–15^ and probes for bioimaging.^16–18^ Semiconductors composed of group 11, group 13, and group 16 elements, popularly termed I–III–VI semiconductors, have been intensively investigated as QD materials,^4,9,10,15,19–22^ because they are composed of less toxic elements and their optical properties are tunable by the kind of elements used, the off-stoichiometric composition, and the formation of a solid solution with other semiconductors as well as by the particle size. For example, the energy gaps (*E*_g_s) of CuInS_2_ (ref. 23) and AgInS_2_ QDs^24^ increased with a decrease of particle size due to the quantum size effect. Alloying CuInS_2_ with ZnS increased the *E*_g_ of the resulting Zn–Cu–In–S solid solution QDs.^25^ QDs composed of a Zn–Ag–In–S solid solution exhibited a broad defect-site photoluminescence (PL) peak, the peak wavelength being blue-shifted from *ca.* 810 to 530 nm with an increase in the Zn fraction.^26^ Recently, we reported Ag-based I–III–VI QDs, such as Ag–In–Ga–S^27^ and Ag–In–Ga–Se,^28^ showing a sharp band-edge PL peak in addition to a broad emission peak when they were prepared with Ag-deficient compositions.

Considerable efforts have been devoted to modifying the optical and electric properties of QDs.^29–33^ Doping of different elements into QDs is one of the useful strategies for tuning their optical properties and improving their functions. For example, doping QDs of ZnS^34^ and Zn–Cd–S^35^ with Mn^2+^ produced an orange emission originating from the doped ions. II–VI semiconductor QDs produced a broad PL peak by Cu^+^ doping as a result of the recombination of delocalized conduction-band electrons with holes trapped at doped Cu^+^ sites.^30^ The peak wavelength of localized surface plasmon resonance (LSPR) of CdO QDs was controlled in the near-IR region by changing the free charge carrier density with co-doping of In^3+^ and F^−^.^36^ The preparation of ZnSe QDs in the presence of Na^+^ ions caused the appearance of an additional PL peak originating from selenium vacancies, accompanied by the increase of the average size from *ca.* 2 to 4 nm.^37^ As for I–III–VI semiconductors, Na^+^ doping was reported to enhance the electric properties of bulk materials. For example, the performance of Cu(In,Ga)Se_2_ thin film solar cells was remarkably improved by the addition of Na^+^ during their preparation because of the efficient growth of crystal grains^38–40^ and/or the increase of carrier density with doped Na^+^ ions,^41–43^ although their origin has not been elucidated and still remains in dispute.^44,45^ Doping AgInSe_2_ with Na^+^ yielded a larger carrier concentration, causing enhancement of its thermoelectric property.^46^ However, there have been few reports on Na^+^ doping in I–III–VI-based QDs.

Surface passivation of QDs with wide-gap semiconductors is another strategy for tuning their optoelectronic properties *via* removal of surface defect sites. For this purpose, ZnS coating was most commonly used for binary II–VI QDs, such as CdS^47–49^ and CdSe,^50–53^ to enhance the PL intensity of a narrow band-edge emission. However, the use of ZnS was not suitable as a shell material for enhancing the band-edge emission of I–III–VI QDs because a broad defect-site PL peak appeared owing to alloy formation between the ZnS shell and the I–III–VI core.^16,54,55^ Recently, we reported that surface coating of Ag–In–Ga–(S,Se) QDs with a GaS_*x*_ shell successfully removed their surface defect sites, followed by the enlargement of a sharp band-edge emission peak.^27,28,56^ By optimizing the preparation conditions, the PL quantum yield (QY) reached 75% for the band-edge emission at 543 nm with Ag–In–Ga–S@GaS_*x*_ core–shell QDs.^57^ Furthermore, it was reported that a Ga–Zn–S alloy shell was also useful for improving the narrow PL peak of Cu–In–Ga–S^58^ and Ag–Ga–S^59^ QDs without broadening their peak width. In contrast, surface coating of Ag–In–S QDs with an InS_*x*_ shell also produced a band-edge emission peak, but a broad PL peak still remained.^55^ Since GaS_*x*_ is less stable under certain conditions, such as the presence of moisture or oxygen,^28,56,60^ there is room for exploring shell materials for practical applications of I–III–VI QDs.

In this study, we used a wide-bandgap Ga_2_O_3_ semiconductor (4.9 eV) for removing surface defect sites on Ag–In–Ga–S solid solution (AIGS) QDs. Heating-up synthesis with the use of thiourea as an S precursor produced AIGS QDs embedded in a Ga_2_O_3_ matrix. The obtained particles simply showed a sharp band-edge PL peak without any post treatment. The PL quantum yield was enhanced from 29% to 58% by the addition of Na^+^ ions to the precursors used for the QD synthesis. To demonstrate the potential capability of thus-obtained composite particles for practical devices, we fabricated QD-light-emitting diodes (QD-LEDs) and evaluated their performance, in which the electroluminescence (EL) peak was similar to that in the PL spectrum of the QDs used.

## Experimental section

### Materials

The chemicals of indium(iii) acetate, gallium(iii) acetylacetonate, and oleylamine (OLA) were purchased from Sigma-Aldrich. Solium acetate, thiourea, and sulfur powder were purchased from Kishida Reagents Chemicals. 1-Dodecanethiol (DDT) was obtained from FUJIFILM Wako Pure Chemical Corp. Molybdenum(vi) oxide and aluminium were bought from Kojundo Chemical Laboratory Co., Ltd. Tris(2,4,6-trimethyl-3-(pyridin-3-yl)phenyl)borane (3TPYMB) and tris(4-carbazoyl-9-ylphenyl)amine (TCTA) were purchased from Luminescence Technology Corp. The content of water in OLA used as a solvent was determined to be *ca.* 600 ppm by the Karl Fisher titration method (Kyoto Electronics Manufacturing, MKC-610).

### Syntheses of AIGS QDs with and without Na^+^ doping

The Na^+^ doping into AIGS QDs was carried out by the synthesis of AIGS QDs in the presence of Na^+^ ions with our previously reported heating-up method^27^ with a slight modification. An Ag-deficient condition was used, in which the ratio of (In + Ga)/(Ag + Na) was 1.5. Typically, mixed powders of 0.060 mmol sodium acetate, 0.14 mmol silver acetate, 0.060 mmol indium acetate, 0.24 mmol gallium acetylacetonate, and 0.55 mmol thiourea were added to a mixture solution of 2.75 cm^3^ OLA and 0.25 cm^3^ DDT. The suspension was heated at 150 °C for 10 min with vigorous stirring under an N_2_ atmosphere, immediately followed by heating at 300 °C for 10 min. After cooling to room temperature, the thus-obtained suspension was subjected to centrifugation to remove aggregated large particles. Target QDs were isolated from the supernatant by adding methanol as a non-solvent, followed by centrifugation. The precipitates were washed several times with ethanol and then dissolved in chloroform. Thus-obtained QDs, prepared with thiourea (tu) as an S precursor, were denoted as Na-AIGS(*R*)(tu) QDs, where *R*, represented by the ratio of (In + Ga)/(Ag + Na) in the precursors, was 1.5. The preparation of Na-AIGS(1.5)(tu) under S-rich conditions also followed the same procedure, with the exception that the amount of thiourea added was increased by 1.5 and 2 times.

For comparison, we prepared another type of Na^+^-doped AIGS QDs by the above-described method except for the use of elemental sulfur (es) powder as an S precursor, and the QDs obtained were denoted as Na-AIGS(1.5)(es) QDs. Furthermore, AIGS QDs were also synthesized in a similar way without the addition of sodium acetate and the QDs obtained from thiourea and elemental sulfur as S precursors were denoted as AIGS(1.5)(tu) and AIGS(1.5)(es) QDs, respectively.

### Syntheses of Na-AIGS(tu) QDs prepared with different metal ratios

We evaluated the influence of the In/Ga and Na/Ag ratios on the PL properties of Na-doped AIGS QDs prepared with thiourea. To avoid complexity, the molar ratios of precursors were varied with the synthesis of stoichiometric Ag_*x*_Na_(1−*x*)_In_*y*_Ga_(1−*y*)_S_2_ QDs in mind, being different from those used for the earlier preparation of Na-AIGS(1.5)(tu) QDs with non-stoichiometric precursor ratios. A metal precursor was mixed powders of sodium acetate, silver acetate, indium acetate, and gallium acetylacetonate under the conditions of (Ag + Na) = (In + Ga) = 0.20 mmol. The metal precursor and 0.40 mmol thiourea were added to a mixture solution of OLA (2.90 cm^3^) and DDT (0.10 cm^3^). The suspension was heated at 150 °C for 10 min with vigorous stirring under an N_2_ atmosphere, immediately followed by heating at 300 °C for 10 min. After cooling to room temperature, target QDs were isolated in a manner similar to that described above. Thus-obtained QDs were denoted as Na-AIGS(1.0)(tu) QDs, considering the stoichiometric condition of (In + Ga)/(Ag + Na) = 1.0 for their preparation.

### Characterization of QDs

The composition of QDs was determined by using energy-dispersive X-ray spectroscopy (EDS, HORIBA Emax Energy EX-250) or X-ray fluorescence spectroscopy (Rigaku NEX CG). The size and shape of QDs were investigated by using a transmission electron microscope (TEM, Hitachi H7650) with an operation voltage of 100 kV. TEM samples were prepared by dropping a small amount of the QD chloroform solution onto a copper TEM grid covered with an amorphous carbon overlayer (Okenshoji Co., Ltd., ELS-C10 STEM Cu100P grid), followed by drying. Using a Cs-corrected HR-STEM (JEOL Co. Ltd, ARM-200F) with an acceleration voltage of 200 kV, we obtained high-resolution images of a high-angle annular dark-field scanning transmission electron microscope (HAADF-STEM). Nanoscale EDS analysis was carried out during the HAADF-STEM measurement. X-ray diffraction (XRD) patterns of particles were obtained with the use of an X-ray diffractometer (Rigaku SmartLab-3K) with Cu Kα radiation.

Absorption spectra were measured with an Agilent 8453A diode array spectrophotometer. A photonic multichannel analyzer (Hamamatsu PMA-12) was used to obtain PL spectra at room temperature with the excitation of 365 nm light. The PL quantum yield (QY) was evaluated with 365 nm light excitation by using an absolute PL quantum yield measurement system (Hamamatsu C9920-03). PL lifetimes were evaluated by measuring PL decay profiles with a time-correlated single-photon counting apparatus (Hamamatsu Quantaurus-Tau) with 365 nm excitation at room temperature. We evaluated the ionization energy with photoemission yield spectroscopy in air (PYSA, Riken Keiki AC-2).

### Fabrication and characterization of QD-LEDs

Our previously reported method^61^ was used for the fabrication of QD-LEDs. An indium tin oxide (ITO) electrode patterned on a glass substrate was cleaned by UV-ozone treatment and used as a substrate. A 40 nm-thick ZnO layer as an electron injection layer (EIL) was prepared by spin-coating an ethanol solution containing ZnO nanoparticles. Subsequently, an emitting layer (EML) was prepared by spin-coating a chloroform solution containing Na-AIGS(1.5)(tu) QDs (5.0 mg cm^−3^) and 3TPYMB (2.5 mg cm^−3^) on the ZnO layer. The molecules of 3TPYMB in the EML worked as an additional electron transporting material (ETM). Furthermore, a hole-transport layer (HTL) of TCTA (thickness: 40 nm), a hole injection layer (HIL) of MoO_3_ (thickness: 10 nm), and an Al anode (thickness: 80 nm) were prepared on the EML in that order by vacuum deposition. A Konica-Minolta CS-2000A spectroradiometer was used to measure the EL spectra of thus-obtained QD-LEDs. The luminance–current–voltage profiles were examined with a luminance meter (Konica Minolta LS-110) and a source meter (Keithley Model 2400). The external quantum efficiencies (EQEs) were evaluated from the current density, luminance, and EL spectrum under the assumption of Lambertian emission.

## Results and discussion

**Fig. 1:**
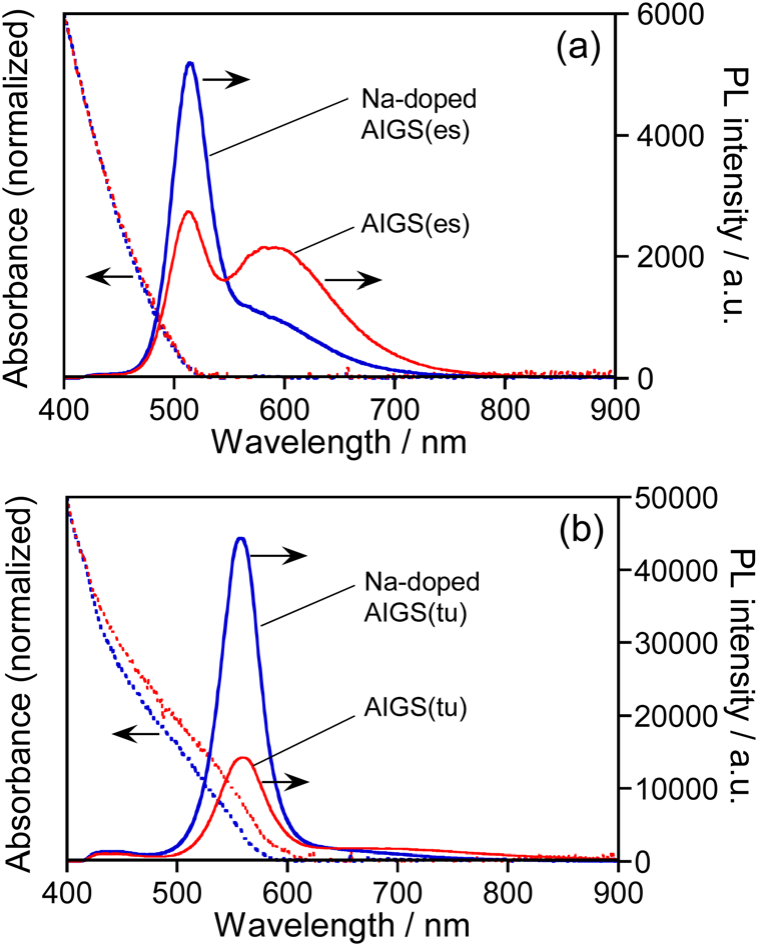
Absorption and PL spectra of AIGS(1.5) QDs prepared with different kinds of S precursors in the absence or presence of Na^+^ ions. The S precursors used were (a) elemental sulfur and (b) thiourea. The wavelength of excitation light for PL measurements was 365 nm. The QDs were prepared with the ratios of Na/(Ag + Na) = 0.30 and In/(In + Ga) = 0.20 in the precursors.

Fig. 1 shows the absorption and PL spectra of QDs prepared with different types of S precursors in the absence or presence of Na^+^ ions. The AIGS QDs prepared with elemental sulfur exhibited similar structureless absorption spectra (Fig. 1a), the absorption onsets of which were 520 nm regardless of Na^+^ addition. The PL spectra of the QDs contained both a sharp peak at 514 nm and a broad peak at *ca.* 590 nm: the former peak was assignable to the band-edge emission and the latter originated from the defect sites. The relative intensity of band-edge emission became larger for the QDs with Na^+^ doping and the PL QY was enlarged from 8.3% for AIGS(1.5)(es) to 12% for Na-AIGS(1.5)(es), indicating that the amount of defect sites in AIGS QDs was decreased by the addition of Na^+^ in the precursors. On the other hand, the AIGS QDs prepared with the use of thiourea exhibited optical properties different from those of AIGS QDs prepared with elemental sulfur. The onset wavelengths of absorption spectra, 590 nm for AIGS(1.5)(tu) and 580 nm for Na-AIGS(1.5)(tu), were longer than those of AIGS(1.5)(es). Only a sharp band-edge PL peak appeared in the PL spectra, regardless of the Na^+^ addition, in which PL peak wavelength was slightly blue-shifted from 560 nm for AIGS(1.5)(tu) to 557 nm for Na-AIGS(1.5)(tu). The PL QY of AIGS(1.5)(tu) was increased from 29% to 58% by the Na^+^ doping, being much larger than that of Na-AIGS(1.5)(es), 12%. These results suggested that the amount of defect sites in AIGS nanocrystals acting as radiative recombination sites became much smaller for the preparation with thiourea than for the preparation with elemental sulfur and that the addition of Na^+^ further reduced the amount of defect sites, causing non-radiative recombination of charge carriers.

As mentioned in the introduction, it was reported that the performance of Cu(In,Ga)Se_2_ thin film solar cells was remarkably improved when the films were grown in the presence of Na^+^ ions. One explanation for the effect of Na^+^ addition was the increase of carrier concentration in the Cu(In,Ga)Se_2_ semiconductor. Using atom probe tomography, Choi *et al.* found that Na^+^ ions doped in Cu(In,Ga)Se_2_ occupied Cu vacancies to produce Na_Cu_ point defects, inhibiting the formation of In_Cu_ antisite defects as a compensating donor and then increasing the effective p-type doping.^62^ Considering the analogy of I–III–VI semiconductors, it was also expected in the present study that Na^+^ ions were doped into Ag-deficient Ag(In,Ga)S_2_ nanocrystals to occupy Ag vacancies that contributed to non-radiative recombination or defect-site emission, resulting in the enhancement of a sharp band-edge emission of AIGS QDs as well as the suppression of a broad emission.

**Fig. 2:**
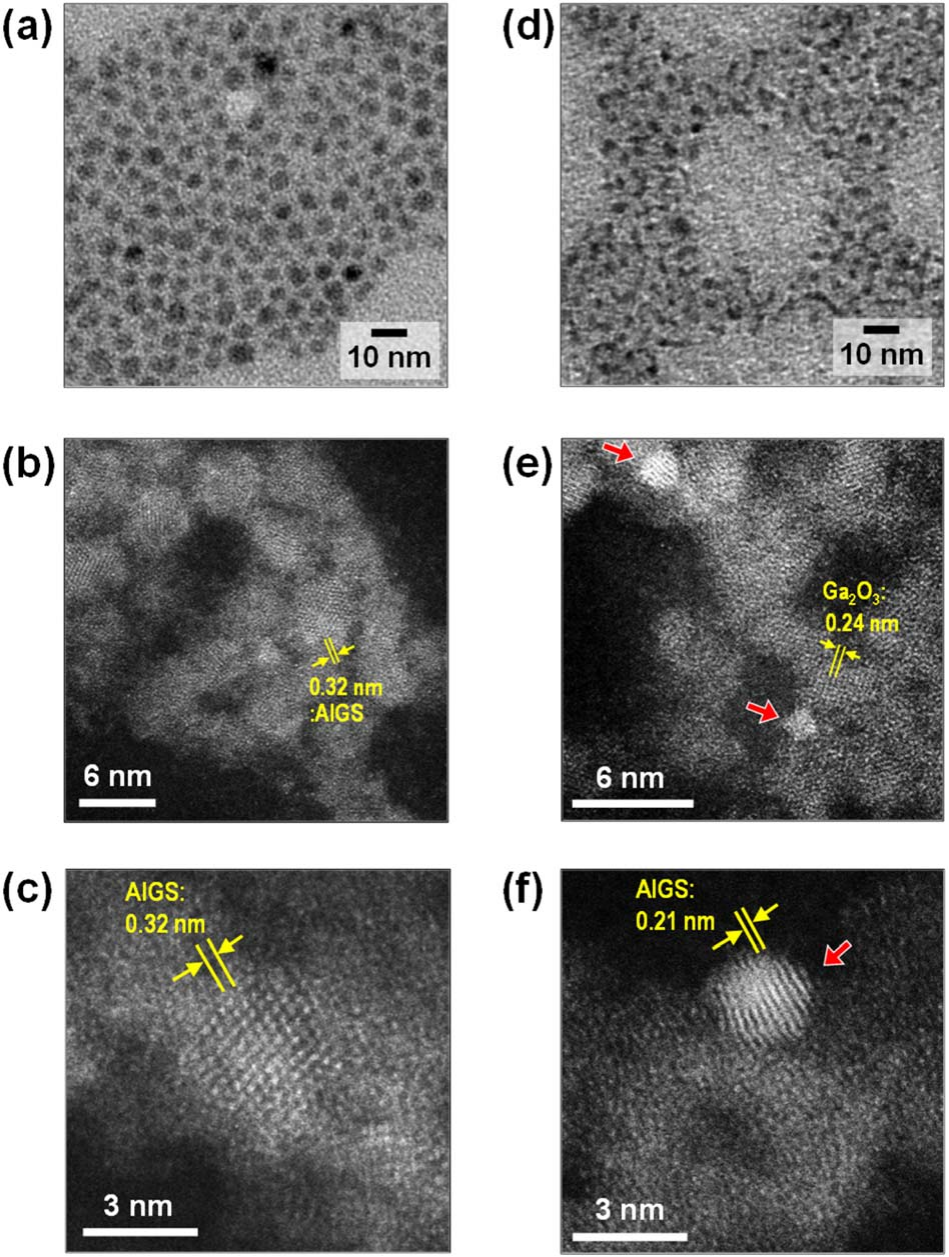
Wide-area TEM images (a and d) and high-resolution HAADF-STEM images (b, c, e and f) of Na-doped AIGS(1.5) QDs. The samples were prepared with elemental sulfur (a–c) and thiourea (d–f). Red arrows in the panels (e) and (f) indicate AIGS nanocrystals embedded in Ga_2_O_3_ matrix showing the lattice fringes with the spacing of 0.24 nm.

Fig. 2 shows TEM images of QDs prepared in the presence of Na^+^ ions. Their size and shape were very similar to those of corresponding QDs prepared without Na^+^ addition, regardless of the type of S precursor (Fig. S1†). In the case of Na-AIGS(1.5)(es) QDs, spherical particles with an average size (*d*_av_) of 4.5 nm and standard deviation (*σ*) of 0.76 nm were observed. High-resolution HAADF-STEM measurements revealed that the QDs exhibited clear lattice fringes with a lattice spacing of 0.32 nm. In contrast, Na-AIGS(1.5)(tu) QDs were composed of irregularly shaped elongated nanocrystals with an average width of 3.4 nm. This change in the particle structure was induced by the replacement of elemental sulfur with thiourea, because AIGS(1.5)(tu) QDs prepared without Na^+^ doping were also composed of similar irregularly shaped nanocrystals (Fig. S1†). HAADF-STEM measurements revealed that Na-AIGS(1.5)(tu) QDs were composed of two types of nanocrystals. Some of them were spherical nanocrystals with sizes of 1.5–2 nm that showed a higher contrast due to the inclusion of heavier atoms, and they exhibited clear lattice fringes with interplanar spacing of 0.21 nm (Fig. 2f), being similar to that of (220) planes of tetragonal AgInS_2_, 0.208 nm. The others were irregularly shaped nanocrystals with a lower contrast, the sizes of which were 3 nm or more, and they had lattice fringes with spacing of 0.24 nm, being assignable to (311) planes of a cubic γ-Ga_2_O_3_ crystal. It should be noted that the particles of the former type were surrounded by those of the latter type, which had a larger number of particles. Nanoscale EDS analysis conducted during HAADF-STEM measurements revealed that nanocrystals with different compositions were contained in Na-AIGS(1.5)(tu) QDs: particles of a lower contrast had the ratio of Ag : In : Ga : S = 0 : 0 : 92.2 : 7.8, suggesting the formation of Na^+^-doped Ga_2_O_3_, whereas those of a higher contrast seemed to be Ag–In–Ga–S nanocrystals embedded in Ga_2_O_3_, showing the composition ratio of Ag : In : Ga : S = 0.7 : 1.7 : 96.2 : 1.4. These findings indicated the formation of Ag–In–Ga–S nanocrystals embedded in a Ga_2_O_3_ matrix (AIGS/Ga_2_O_3_ composites). It should be noted that the Na fraction could not be determined during HAADF-STEM measurements because of the superimposition of signals between Na and Ga in the nanoscale EDS analysis.

**Fig. 3:**
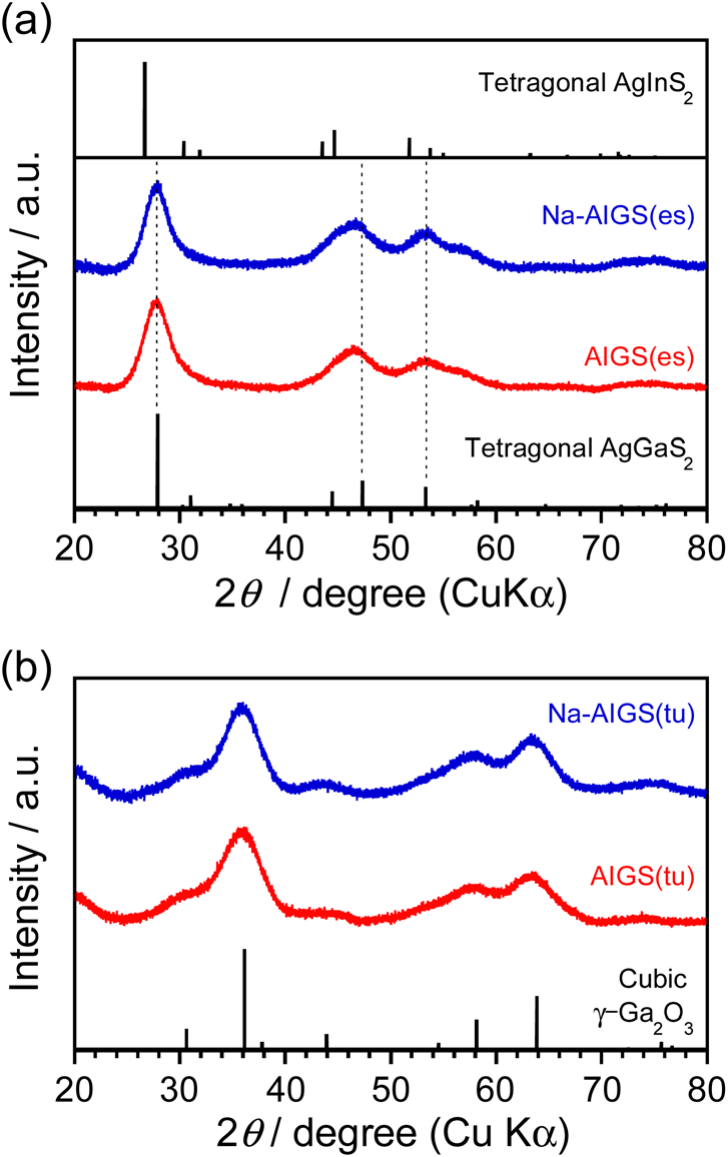
XRD patterns of AIGS(1.5) QDs prepared with (a) elemental sulfur and (b) thiourea as an S precursor. The samples were prepared under the same conditions as those in Fig. 1.

As shown in Fig. 3a, each diffraction peak of AIGS(1.5)(es) and Na-AIGS(1.5)(es) QDs was between the corresponding peaks of tetragonal AgGaS_2_ and tetragonal AgInS_2_ crystal structures, indicating the formation of an Ag–In–Ga–S solid solution regardless of Na^+^ addition. The In/(In + Ga) ratios were estimated from elemental analysis (Table 1) to be 0.21 and 0.26 for AIGS(1.5)(es) and Na-AIGS(1.5)(es) QDs, respectively, being in rough agreement with that expected from the ratio of metal precursors used, 0.20. The experimentally obtained Na fraction of Na^+^-AIGS(1.5)(es) QDs, Na/metal = 0.064, being much lower than that used in the precursors, 0.12. The charge ratios of anion/cation estimated from the results shown in Table 1 were 1.1 for AIGS(1.5)(es) and 0.93 for Na-AIGS(1.5)(es) QDs, suggesting that the AIGS(1.5)(es) QDs were composed of an Ag–In–Ga–S solid solution without containing a significant amount of by-product crystal phases such as metal oxides.

**Table 1:** Chemical compositions of AIGS(1.5) QDs prepared with and without Na^+^ addition

Samples^a^	Fraction (%)					Charge balance (anion/cation)	Metal ratios	
	Na	Ag	In	Ga	S		In/(In + Ga)	Na/metal
AJGS(es)	—	16	7.3	21	56	1.1	0.26	0
Na-AIGS(es)	3.1	15	6.6	24	51	0.93	0.21	0.064
AIGS(tu)	—	0.29	0.28	76	24	0.24	0.004	0
Na-AIGS(tu)	2.5	1.2	0.67	70	26	0.21	0.01	0.034

a The samples were prepared under the same conditions as those in Fig. 1.

On the other hand, both AIGS(1.5)(tu) and Na-AIGS(1.5)(tu) QDs exhibited broad XRD patterns, in which each broad diffraction peak was assignable to that of cubic γ-Ga_2_O_3_ crystal phase, but peaks originating from the Ag–In–Ga–S solid solution were not detected (Fig. 3b). These results indicated that a large amount of Ga_2_O_3_ phase was contained in the QDs prepared with thiourea, being consistent with the observation in the HAADF-STEM image. Elemental analysis revealed that the Ga fraction in the QDs was 70 at% or more, being much larger than that in the precursors, and then the sums of fractions of Ag and In were very small, *ca.* 0.57% and 1.9% in AIGS(1.5)(tu) and Na-AIGS(1.5)(tu), respectively (Table 1). Furthermore, it should be noted that the S fractions of AIGS(1.5)(tu) were about half those of AIGS(1.5)(es) and that the charge ratios of anion/cation of AIGS(1.5)(tu) were considerably less than 1, regardless of Na^+^ doping. The results of XRD patterns and elemental analysis suggested that a large amount of oxygen atoms was present as an anionic species, probably as O^2−^, in addition to S^2−^ in the samples of AIGS(1.5)(tu) and Na-AIGS(1.5)(tu). Thus, we can conclude for the QDs prepared with thiourea that the incorporation of Ag–In–Ga–S nanocrystal cores in the Ga_2_O_3_ matrix effectively removed their surface defect sites, which caused a broad PL peak or contributed to non-radiative recombination.

We also carried out the preparation of AIGS(tu) QDs under S-rich conditions. Even when the quantity of thiourea added was increased by 1.5 and 2 times without changing the amount of metal precursors, the chemical composition of the resulting Na-AIGS(1.5)(tu) was not significantly changed: the S fraction slightly increased from 26% to 34% with an increase in the thiourea addition from 0.55 mmol to 1.1 mmol (Table S1†). However, as shown in Fig. S2,† the resulting QDs exhibited a blue shift of the band-edge PL peak along with the shift in absorption spectra. It should be noted that the PL QY of Na-AIGS(1.5)(tu) was remarkably deteriorated from 58% to 18% with an increase in the thiourea addition (Table S2†). This phenomenon was probably attributed to an increase in the number of surface defect sites on AIGS nanocrystal cores, caused by a decrease in their size from 3.4 nm to 3.0 nm (Fig. 2 and S2†).

**Fig. 4:**
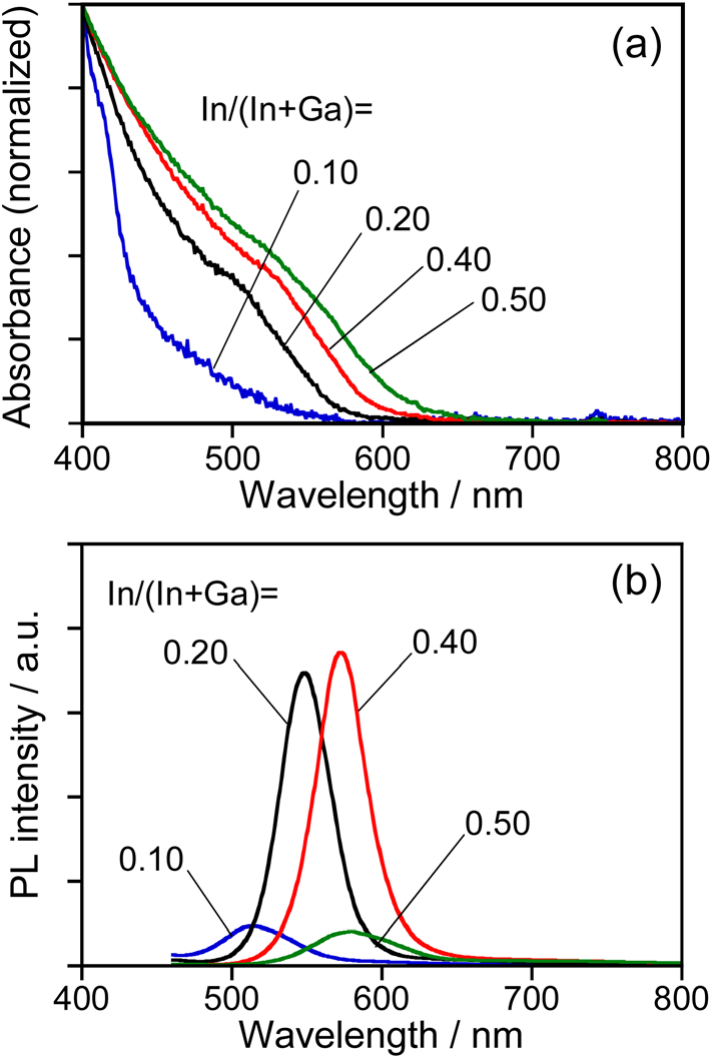
Absorption (a) and PL (b) spectra of Na-AIGS(1.0)(tu) QDs prepared with different In/(In + Ga) ratios under the fixed ratio of Na/(Ag + Na) = 0.30 in the precursors. The numbers in the figure represent the In/(In + Ga) ratios in the precursors.

As reported in our previous paper,^27^ the QDs composed of an Ag–In–Ga–S solid solution exhibited composition-dependent optical properties: *E*_g_ decreased with an increase in the In fraction, resulting in a red shift of the band-edge PL peak. In order to confirm the formation of Ag–In–Ga–S solid solution, therefore, we evaluated the tunability of optical properties of Na-AIGS(tu) QDs with the In fraction in the precursors under the fixed ratio of (In + Ga)/(Ag + Na) (=*R*) of 1.0. Regardless of the In fraction, the resulting Na-AIGS(1.0)(tu) QDs exhibited XRD patterns assignable to cubic γ-Ga_2_O_3_ crystal (Fig. S3†) and contained a large Ga fraction in their compositions of >60% (Table S3†). The average size was increased from 3.2 nm to 6.1 nm with an increase of In/(In + Ga) ratio from 0.10 to 0.50 (Fig. S4†). Fig. 4 shows the absorption and PL spectra of Na-AIGS(1.0)(tu) QDs prepared with different In/(In + Ga) ratios in the precursors. With an increase in the In/(In + Ga) ratio from 0.10 to 0.50, the absorption onset was red-shifted from *ca.* 540 nm to 610 nm, indicating that *E*_g_ decreased from 2.3 eV to 2.0 eV due to the increase in the In fraction of Ag–In–Ga–S nanocrystal cores. The Na-AIGS(1.0)(tu) QDs exhibited a band-edge PL peak without showing a broad peak, and the PL peak wavelength was shifted from 513 nm to 579 nm with an increase in the In fraction. These results indicated that the Ag–In–Ga–S nanocrystals had a controllable composition with the metal precursor ratio, In/Ga, although they were incorporated in the Ga_2_O_3_ matrix of Na-AIGS(tu) QDs. The values of PL QY of Na-AIGS(1.0)(tu) QDs in Fig. 4b are shown in Table S4.† The PL QY became larger for the QDs prepared with In/(In + Ga) = 0.20–0.40. Similar behavior was observed for Ag–In–Ga–S,^27^ Ag–In–Ga–Se,^28^ and Cu–In–Ga–S^58^ QDs. Although the precise reason why the In fraction affected the PL QY of QDs remains unclear, it is plausible that the crystallinity of Ag–In–Ga–S cores was influenced by the In/(In + Ga) ratio in the precursors, resulting in the variation in the amount of non-radiative defect sites. It should be noted that the PL QY of resulting Na-AIGS(tu) QDs was decreased from 58% (Fig. 1b, Table S2†) to 36% (Fig. 4b, Table S4†) with a decrease of the ratio of (In + Ga)/(Ag + Na) from 1.5 to 1.0, even when the preparation was carried out with the fixed ratio of In/(In + Ga) = 0.20. This suggested that non-radiative defect sites were remarkably dependent on the ratio of group-III elements (In and Ga) to group-I element (Ag), being similar to previously reported Ag–In–Ga–S QDs.^27^

The PL property of AIGS QDs prepared with thiourea were also influenced by the Na fraction in the precursors, as shown in Fig. S5.† The amount of Na^+^ doping was slightly increased from 4.4% to 6.0% with an increase in the Na/(Ag + Na) ratio from 0.30 to 0.70 (Table S5†). The absorption spectra of Na-AIGS(1.0)(tu) QDs were red-shifted and their PL QY was significantly deteriorated from 36% to 0.6% (Table S6†) by increasing the Na/(Ag + Na) ratio from 0.30 to 0.70, because of the increase of particle size from 3.9 (Fig. S4b†) to 12 nm (Fig. S5c†), respectively.

Since it was difficult to experimentally determine the chemical composition of Ag–In–Ga–S nanocrystals for AIGS(1.5)(tu) QDs due to the presence of a large amount of Ga_2_O_3_ particles, we roughly estimated the In fraction from the band-edge PL peak by assuming that the previously reported relation between the band-edge PL peak position and the In/(In + Ga) ratio of AIGS QDs^27^ was applicable to the present case. From the wavelength of the band-edge PL peak at 557 nm for Na-AIGS(1.5)(tu) QDs prepared with In/(In + Ga) = 0.20 (Fig. 1b), Ag–In–Ga–S nanocrystals in the Ga_2_O_3_ matrix were presumed to have the composition of In/(In + Ga) = 0.7, being much larger than that of Na-AIGS(1.5)(es) QDs, 0.21. Thus, in the case of using thiourea, a large fraction of Ga in metal precursors was not used for formation of the Ag–In–Ga–S nanocrystal phase but was deposited as a Ga_2_O_3_ matrix surrounding the nanocrystals, where *ca.* 600 ppm water, included as an impurity in OLA, probably served as an oxygen source for Ga_2_O_3_. It was reported that γ-Ga_2_O_3_ particles were produced by simply heating Ga(acac)_3_ in OLA at a temperature of 200 °C or higher.^63^ Furthermore, it seemed likely that the reactivity of thiourea with Ga^3+^ was lower than that of S powders. Therefore, the formation rate of Ag–In–Ga–S nanocrystal cores was considered to relatively slow with the use of thiourea, and then thermolysis of Ga(acac)_3_ in OLA proceeded as a side reaction, resulting in the formation of a γ-Ga_2_O_3_ crystal phase surrounding Ag–In–Ga–S nanocrystal cores.

**Fig. 5:**
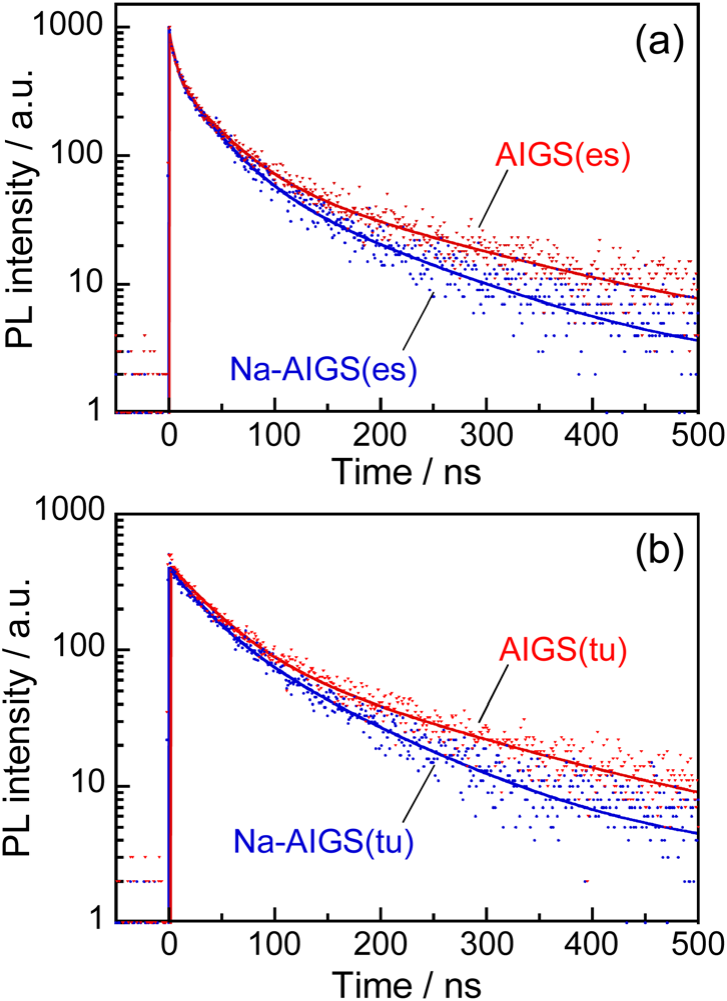
PL decay profiles of (a) AIGS(1.5)(es) and (b) AIGS(1.5)(tu) QDs prepared with or without Na^+^ addition. The experimentally obtained results were fitted by three- or two-component exponential curves (solid lines) with the parameters listed in Table S7.† The samples were prepared under the same conditions as those in Fig. 1.

Fig. 5 shows the PL decay profiles of AIGS QDs. Regardless of the kind of S precursors, the Na^+^ doping slightly accelerated the rate of PL decay. The profiles for the QDs prepared with elemental sulfur and thiourea were fitted well with three- and two-component exponential equations (eqn (1)), respectively.1
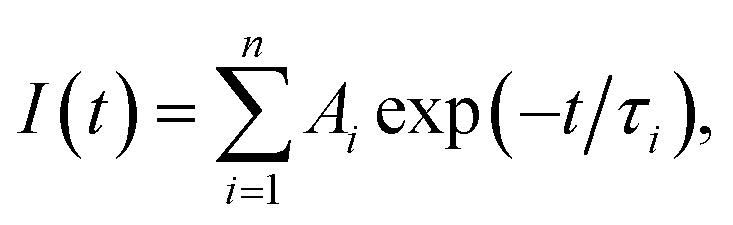
where *τ*_*i*_ is a decay lifetime and *A*_*i*_ is its amplitude. The obtained parameters are listed in Table S7.† The average lifetime, *τ*_ave_, was calculated by eqn (2).2
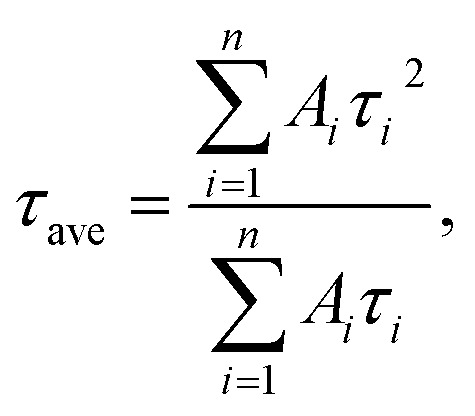


Regardless of the type of AIGS QDs, the average lifetimes were determined to be 70–120 ns, being on the same level as the lifetimes of the band-edge emission from GaS_*x*_-coated AgInS_2_ (ref. 27 and 55) and GaS_*x*_-coated Ag(In,Ga)S_2_.^57,64^ Furthermore, the Na^+^ doping decreased their average PL lifetimes by *ca.* 30%. Considering the increase of the PL QY with Na^+^ doping (Fig. 1), the change in the average lifetime seemed to be caused by the increase of radiative recombination rates in AIGS QDs. It was previously reported^63^ that Ga_2_O_3_ particles of 6 nm in size had the absorption onset at *ca.* 300 nm and exhibited a broad defect-site PL peak at *ca.* 460 nm originating from donor–acceptor pair recombination, in which their PL lifetime was 5670 ns, being about two orders of magnitude longer than that of Na-AIGS(1.5)(tu) QDs in the present study. Thus, these results also supported the assignment that the absorption and PL spectra of Na-AIGS(1.5)(tu) (Fig. 1b) were derived from embedded Ag–In–Ga–S nanocrystals but not from the Ga_2_O_3_ particle matrix.

As mentioned above, it was found that the incorporation of Ag–In–Ga–S nanocrystal cores into the Ga_2_O_3_ matrix was effective for passivating the surface defect sites and furthermore that the Na^+^ doping enlarged the PL QY of band-edge emission. Next, we evaluated the potential of such QDs as an emitting layer (EML) of QD-LEDs. The energy structure of QDs is important information for fabricating QD-LEDs. The levels of the conduction band minimum (CBM) and valence band maximum (VBM) of AIGS QDs were experimentally evaluated by measuring their ionization energy and *E*_g_. The VBM level was assumed to be the opposite sign of the ionization energy of semiconductors and the CBM was estimated by subtracting the *E*_g_ value from the VBM. Similar ionization energies were obtained: 5.47 eV for Na-AIGS(1.5)(tu) and 5.49 eV for AIGS(1.5)(tu) (Fig. S6†). The *E*_g_ values were determined to 2.21 eV for Na-AIGS(1.5)(tu) and 2.15 eV for AIGS(1.5)(tu) from Tauc plots of the absorption spectra. Thus, the VBM and CBM levels of Na-AIGS(1.5)(tu) were estimated to −5.47 and −3.26 eV, respectively, being similar to those of GaS_*x*_-coated Ag(In,Ga)S_2_ QDs.^65^ It should be noted that the energy levels of AIGS(1.5)(tu) QDs were not significantly varied by the Na^+^ doping (Fig. S6e†).

**Fig. 6:**
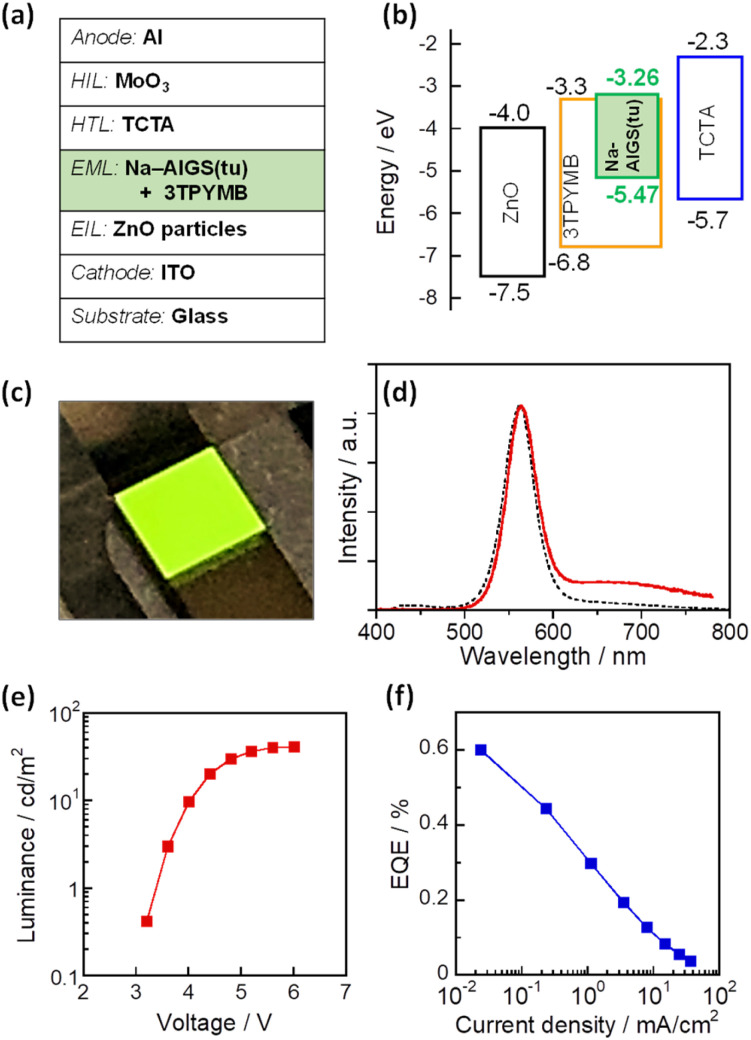
Schematic illustrations of the device structure (a) and the electronic energy structure (b) of a QD-LED fabricated with Na-doped AIGS(tu) QDs. (c) A photograph of the device during the operation. (d) EL spectrum (red solid line) of the obtained QD-LEDs at the luminance of 1.5 cd m^−2^. A PL spectrum (black dotted line) of the corresponding QDs in chloroform is also shown (wavelength of excitation light: 365 nm). Curves of (e) luminance–voltage and (f) EQE-current density of the device.

The QD-LED was fabricated with Na-AIGS(1.5)(tu) QDs prepared with In/(In + Ga) = 0.20 and Na/(Ag + Na) = 0.30 because of their unimodal PL peak with a high PL QY (58%). The structure and energy levels of the device are shown in Fig. 6a and b. The energy levels of ZnO,^66^ TCTA,^67,68^ and 3TPYMB^69^ are literature values. Na-AIGS(1.5)(tu) QDs were mixed with 3TPYMB acting as an electron transporting material and deposited as an EML on the EIL composed of ZnO nanoparticles, in which electron injection seemed to easily occur from 3TPYMB to the QDs due to the similarity between the lowest unoccupied molecular orbital (LUMO) level of 3TPYMB and the CBM of the QDs used. On the EML, TCTA and MoO_3_ layers were successively deposited as HILs. Since 3TPYMB has the highest occupied molecular orbital (HOMO) level much lower than those of the VBM of the QDs and the HOMO of the TCTA layer, the direct injection of holes from the TCTA layer to the ZnO layer is expected to be blocked with 3TPYMB in the EML.

Fig. 6c shows a photograph of the device during the operation and Fig. 6d shows the EL spectrum of the obtained QD-LED at relatively low luminance (1.5 cd m^−2^). An intense sharp EL peak was observed at 563 nm, accompanied by a weak broad EL peak around 700 nm. The former peak had a peak wavelength and peak width close to those of the band-edge PL peak of the Na-AIGS(1.5)(tu) QDs used. In contrast, the broad EL peak was assignable to defect-site emission, although the QDs used hardly exhibited a broad PL peak originating from defect sites. The materials used other than the QDs did not emit light in the wavelength range of 600–800 nm. For multinary QD-based LEDs that we previously reported,^58,61,65^ it was thought that some of the externally injected carriers were trapped at intragap states on the QD surface to generate a broad EL peak and that some of them radiatively recombined between the CBM and VBM levels to form a sharp band-edge EL peak. This was true of the present case.

As shown in Fig. 6e, the luminance of the prepared device increased with an increase in the applied voltage and became a plateau at 5.6 V or larger. The turn-on voltage of the device was observed at *ca.* 3.2 V, being slightly larger than those reported for QD-LEDs fabricated with AIGS@GaS_*x*_ core–shell QDs, 2.2–2.6 V.^65^ Considering the larger *E*_g_ of Ga_2_O_3_ (4.9 eV)^63^ than that of Ga_2_S_3_ (2.6 eV), these results suggested that the Ga_2_O_3_ matrix of Na-AIGS(1.5)(tu) QDs formed a larger energy barrier for the external injection of charge carriers to Ag–In–Ga–S nanocrystals than that of GaS_*x*_ shell layer of AIGS@GaS_*x*_ core–shell QDs. Fig. S7† shows EL peak spectra at different applied voltages. Although the peak profile originating from the QDs was unchanged, an additional emission peak, attributed to the emission from 3TPYMB in the EML, became visible in the wavelength range from 400 to 500 nm, and its relative intensity was enlarged with an increase in the voltage. The observed phenomenon was probably due to the deceleration of carrier injection from 3TPYMB into the Ag–In–Ga–S nanocrystal cores, caused by the presence of Ga_2_O_3_ shells with the large *E*_g_. This resulted in an accumulation of charge carriers within the EML, leading to charge recombination in 3TPYMB. Consequently, the stability of EL intensity was insufficient to accurately assess the device lifetime of the prepared QD-LED. On the other hand, EQE monotonously decreased with an increase in the current density (Fig. 6f). The maximum value of EQE was *ca.* 0.6% at the lowest current density, being comparable to those previously reported for I–III–VI-based QD-LEDs showing a band-edge EL peak, 0.54% with GaS_*x*_-coated AgInS_2_ (ref. 61) and 1.1% with GaS_*x*_-coated Ag(In,Ga)S_2_ QDs.^65,70^ Thus, we can conclude that Ag–In–Ga–S nanocrystals embedded in a Ga_2_O_3_ matrix are applicable to the EML in QD-LEDs, though the performance must be improved by controlling several parameters, including the fraction of Na^+^ doped in QDs, the ratio of Ag–In–Ga–S nanocrystals to the Ga_2_O_3_ matrix, the device structure, and/or the selection of materials for carrier injection layers.

## Conclusions

We developed a one-pot synthesis strategy for Ag–In–Ga–S nanocrystals embedded in Ga_2_O_3_ matrix. The selection of an S precursor was an important factor for controlling the structure of the resulting QDs: The use of elemental sulfur simply produced AIGS QDs without the formation of detectable by-products, while one-pot synthesis using thiourea produced AIGS/Ga_2_O_3_ composite particles, the structure of which was Ag–In–Ga–S nanocrystal cores embedded in a Ga_2_O_3_ matrix. The as-prepared AIGS/Ga_2_O_3_ composite particles showed only a sharp band-edge emission peak without peaks assignable to broad defect-site PL, suggesting that radiative defect sites were scarcely formed at the interface between Ag–In–Ga–S nanocrystals and the Ga_2_O_3_ matrix. Thus, Ga_2_O_3_ is another choice of promising materials for surface coating of I–III–VI QDs without compromising their sharp PL emission peak.

We successfully found the effect of Na^+^ doping on the optical properties of AIGS QDs. Regardless of the kind of S precursor used, elemental analysis revealed that the obtained QDs were doped with Na^+^ ions, the amount of which was *ca.* 3 atom% in the particles. The Na^+^ doping caused a remarkable change in PL spectra: A sharp band-edge PL peak was enlarged due to the prevention of the formation of non-radiative defect sites in Ag–In–Ga–S nanocrystals or on their surface. The maximum PL QY was observed to be 58% for Na-doped AIGS/Ga_2_O_3_ composite particles (Na-AIGS(1.5)(tu)) without further post-synthetic treatment. In contrast, the *E*_g_ of AIGS QDs and their levels of CBM and VBM were not significantly modified by the Na^+^ doping.

The potential of AIGS/Ga_2_O_3_ composite particles for an emitting layer of QD-LEDs was evaluated. Green EL was observed, in which a sharp EL peak appeared at 563 nm, the peak position and width being similar to those of the PL peak of the corresponding QDs. Despite the higher *E*_g_ of Ga_2_O_3_ than that of GaS_*x*_, the turn-on voltage of the prepared device was slightly larger than those reported for AIGS@GaS_*x*_ core–shell QDs. It was shown that Ga_2_O_3_ matrix surrounding AIGS QDs did not significantly prevent the injection of charge carriers to the QDs. Our findings will be important for improving the performance of I–III–VI QDs and for developing their novel applications.

## Author contributions

M. T., S. K., and T. T. conceived the idea to improve the luminescent properties of QDs by Na^+^ doping and designed the project. M. T., C. M., K. A., T. K., T. Y. and T. T. prepared and characterized the QDs. G. M., Y. F., T. U. and S. K. fabricated QD-LEDs and evaluated their performance. M. T. and T. T. wrote the paper. All authors discussed the results and commented on the manuscript.

## Conflicts of interest

There are no conflicts to declare.

## Supplementary Material

NA-005-D3NA00755C-s001
