# Supplementary material for: One-pot synthesis of Ag–In–Ga–S nanocrystals embedded in a Ga2O3 matrix and enhancement of band-edge emission by Na+ doping
Source: Nanoscale Adv. 2023 Nov 14;5(24):7057–66. doi: 10.1039/d3na00755c (PMC10696949; doi:10.1039/d3na00755c)
Supplement: NA-005-D3NA00755C-s001 [file NA-005-D3NA00755C-s001.pdf]

Electronic Supplementary Material (ESI) for Nanoscale Advances.
This journal is © The Royal Society of Chemistry 2023

Supplementary Information

One-pot synthesis of Ag–In–Ga–S nanocrystals embedded in a Ga_2O_3 matrix and enhancement of band-edge emission by Na^+ doping

Makoto Tozawa,[a] Chie Miyamae,[a] Kazutaka Akiyoshi,[a] Tatsuya Kameyama,[a] Takahisa Yamamoto,[a] Genichi Motomura,[b, c] Yoshihide Fujisaki,[b] Taro Uematsu,[c,d] Susumu Kuwabata[c,d] and Tsukasa Torimoto*[a]

[a] Graduate School of Engineering, Nagoya University, Chikusa-ku, Nagoya 464-8603, Japan

[b] Science & Technology Research Laboratories, Japan Broadcasting Corporation (NHK), 1-10-11 Kinuta, Setagaya-ku, Tokyo 157-8510, Japan

[c] Department of Applied Chemistry, Graduate School of Engineering, Osaka University, Suita, Osaka 565-0871, Japan

[d] Innovative Catalysis Science Division, Institute for Open and Transdisciplinary Research Initiatives (ICS-OTRI), Osaka University, Suita, Osaka 565-0871, Japan

* The author to whom correspondence should be addressed:

torimoto@chembio.nagoya-u.ac.jp

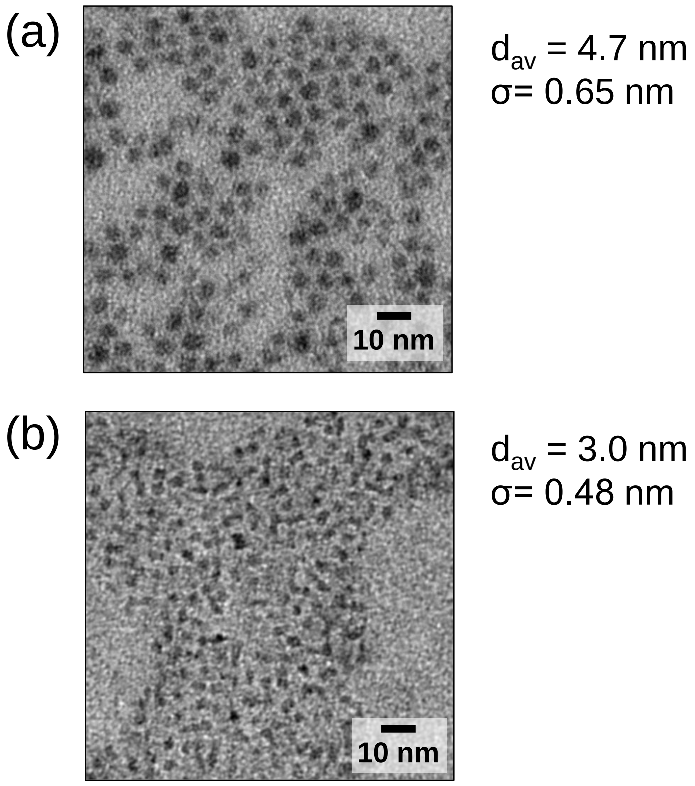

Figure S1. Wide-area TEM images of AIGS QDs prepared in the absence of Na^{+} ions. The samples were prepared with elemental sulfur (a) and thiourea (b) under the ratios of Na/(Ag+Na)= 0.30 and In/(In+Ga)= 0.20 in the precursors. The average size (d_{av}) and standard deviation (σ) of particles are shown beside the corresponding images.

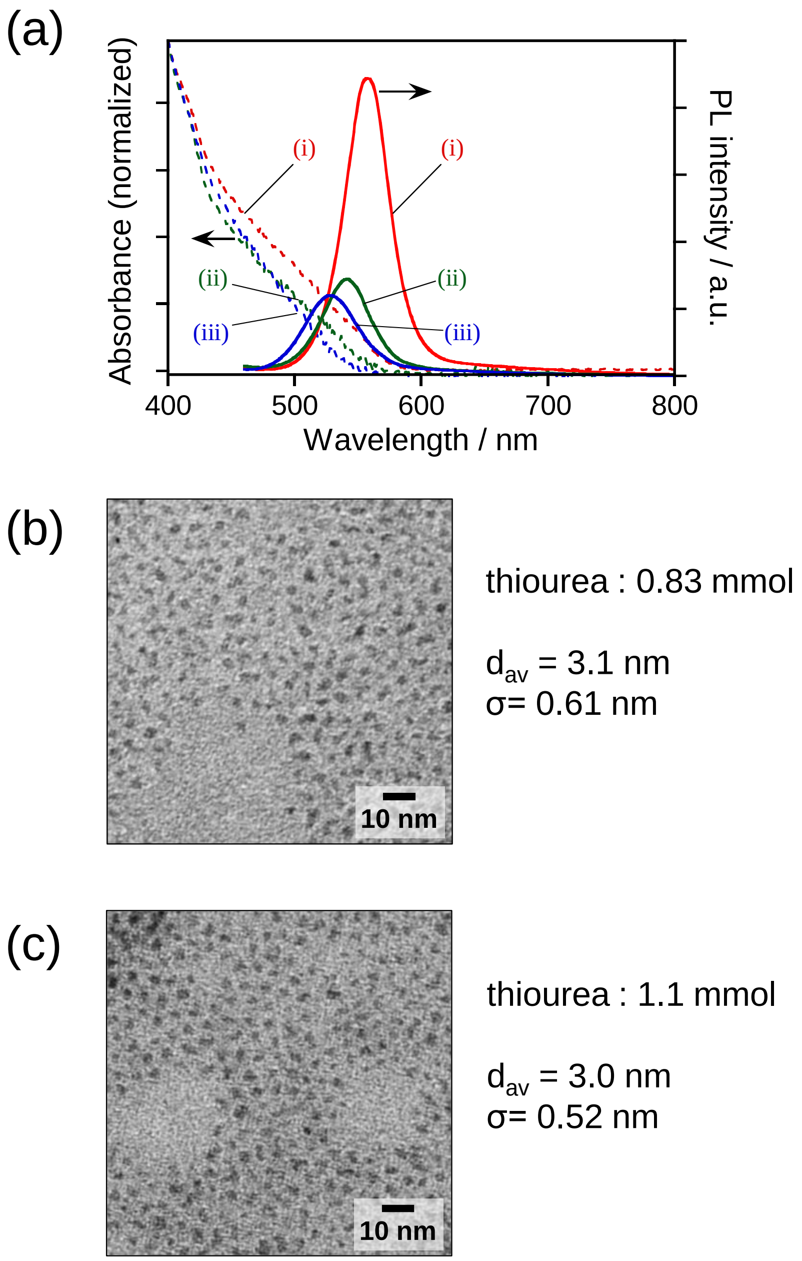

Figure S2. (a) Absorption and PL spectra of Na-AIGS(1.5)(tu) QDs prepared with various amount of thiourea. The QDs were prepared with the addition of (i) 0.55, (ii) 0.83, and (iii) 1.1 mmol thiourea under the fixed ratios of Na/(Ag+Na)= 0.30 and In/(In+Ga)= 0.20 in the precursors. The wavelength of excitation light for PL measurements was 365 nm. (b, c) TEM images of Na-AIGS(1.5)(tu) QDs obtained with (b) 0.83 and (c) 1.1 mmol thiourea in the precursors. The average size (d_{av}) and standard deviation (σ) of particles are shown beside the corresponding images.

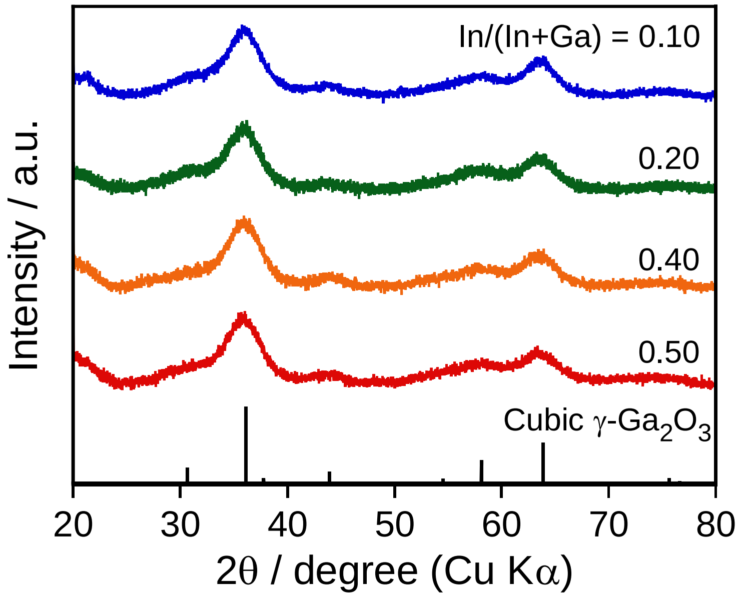

Figure S3. XRD patterns of Na-AIGS(1.0)(tu) QDs with different ratios of In/(In+Ga) in the precursors. The samples were prepared under the same conditions as those in Fig. 4.

(a)

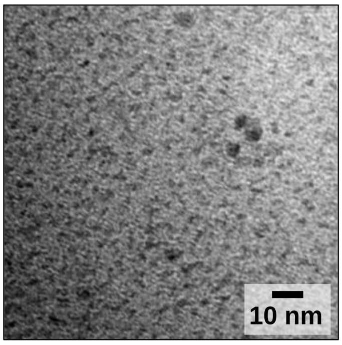

In/(In+Ga) = 0.10
d_{av} = 3.2 nm
σ= 1.2 nm

(b)

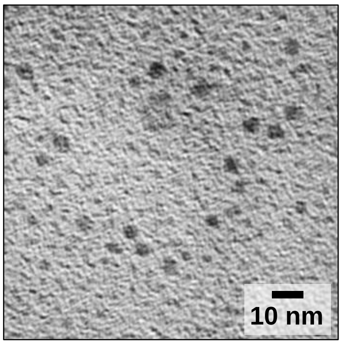

In/(In+Ga) = 0.20
d_{av} = 3.9 nm
σ= 0.76 nm

(c)

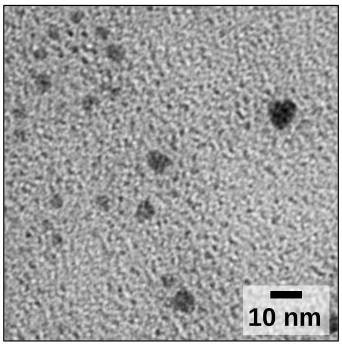

In/(In+Ga) = 0.40
d_{av} = 5.1 nm
σ= 1.4 nm

(d)

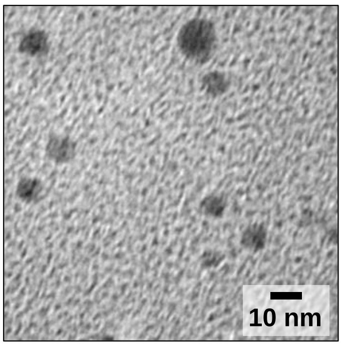

In/(In+Ga) = 0.50
d_{av} = 6.1 nm
σ= 1.8 nm

Figure S4. TEM images of Na-AIGS(1.0)(tu) QDs with different ratios of In/(In+Ga) in the precursors. The average size (d_{av}) and standard deviation (σ) of particles are shown below the corresponding images. The samples were prepared under the same conditions as those in Fig. 4.

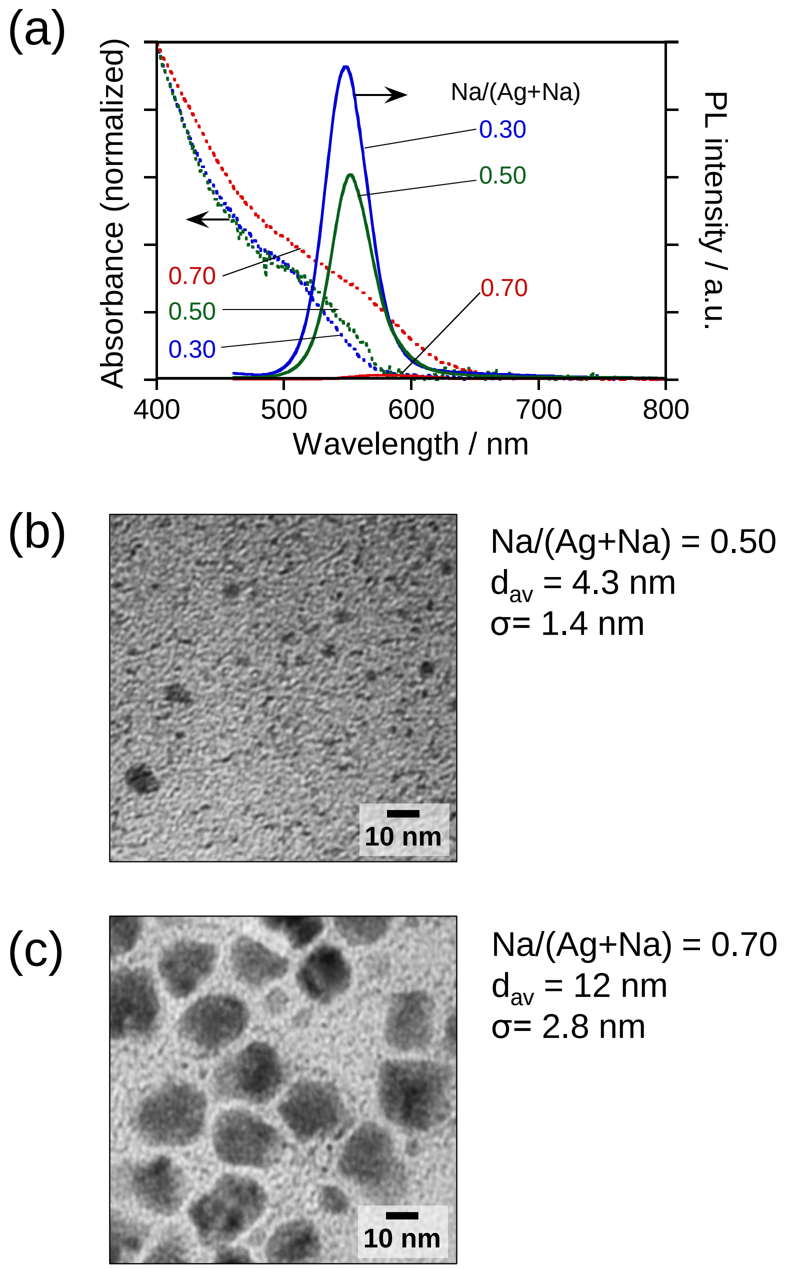

Figure S5. (a) Absorption and PL spectra of Na-AIGS(1.0)(tu) QDs prepared with different Na/(Ag+Na) ratios in the precursors. The wavelength of excitation light for PL measurements was 365 nm. (b, c) TEM images of Na-AIGS(1.0)(tu) QDs with Na/(Ag+Na) of (b) 0.50 and (c) 0.70. The In/(In+Ga) ratio in the precursors was fixed to 0.20.

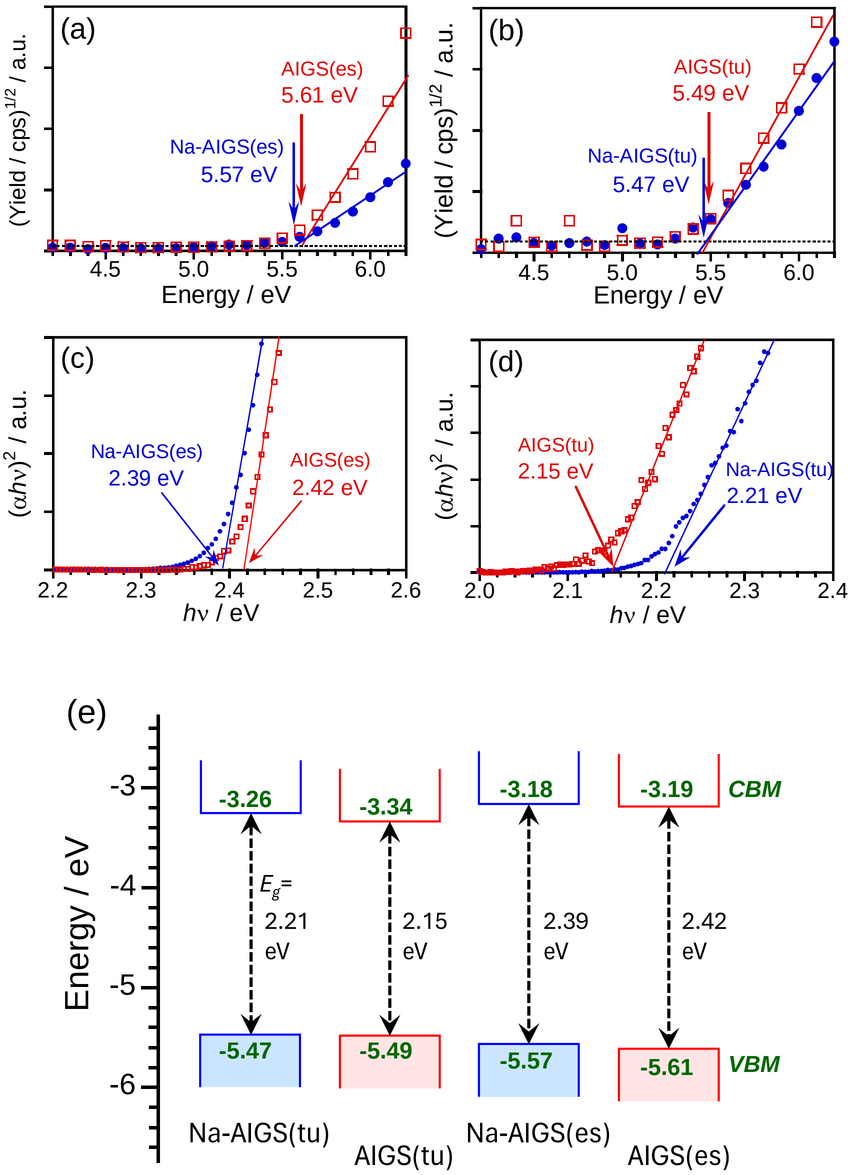

Figure S6. (a, b) Photoelectron yield spectra and (c, d) Tauc plots of AIGS(1.5) QDs prepared with and without Na^+ addition. The S precursors used for the preparation were thiourea (a, c) and elemental sulfur (b, d). (e) Electronic energy structures of AIGS and Na-AIGS QDs. The samples were prepared under the same conditions as those in Fig. 1.

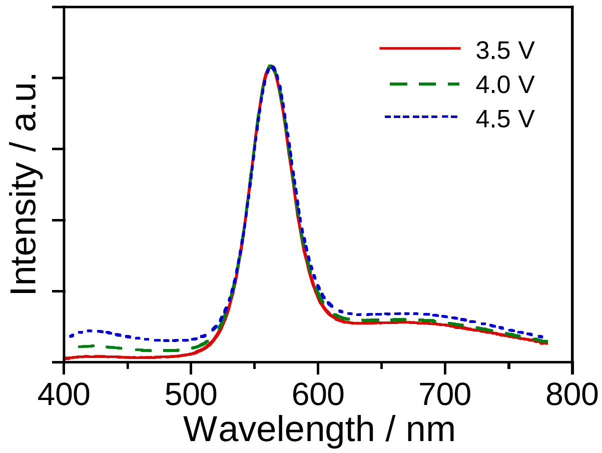

Figure S7. EL spectra of the QD-LED devise containing Na-AIGS(1.5)(tu) QDs at different applied voltages.

Table S1. Chemical compositions of Na-AIGS(1.5)(tu) QDs[(a)] prepared with different amount of thiourea in the precursors.

Amount of thiourea / mmol	Fraction (%)					Charge balance (anion/cation)	Metal ratios	
	Na	Ag	In	Ga	S		In/(In+Ga)	Na/metal
0.55	2.5	1.2	0.7	70	26	0.24	0.01	0.034
0.83	5.0	0.2	0.4	62	33	0.34	0.006	0.074
1.1	5.0	0.5	0.3	60	34	0.36	0.005	0.075

(a) The samples were prepared under the same conditions as those in Fig. S2.

Table S2. PL peak wavelengths and quantum yields of Na-AIGS(1.5)(tu) QDs[(a)] prepared with different amount of thiourea in the precursors.

Amount of thiourea / mmol	Peak wavelength / nm	PL QY / %
0.55	548	58
0.83	541	20
1.1	528	18

(a) The samples were prepared under the same conditions as those in Fig. S2.

Table S3. Chemical compositions of Na-AIGS(1.0)(tu) QDs[(a)] prepared with different In/(In+Ga) ratios in the precursors.

In/(In+Ga) in the precursors	Fraction (%)					Charge balance (anion/cation)	Metal ratio
	Na	Ag	In	Ga	S		In/(In+Ga)
0.10	5.7	0.2	0.2	65	29	0.29	0.002
0.20	4.4	1.3	0.7	63	30	0.30	0.011
0.40	4.4	1.3	1.2	62	31	0.32	0.019
0.50	3.1	0.8	1.7	62	32	0.33	0.027

(a) The samples were prepared under the same conditions as those in Fig. 4.

Table S4. PL peak wavelengths and quantum yields of Na-AIGS(1.0)(tu) QDs[(a)] prepared with different In/(In+Ga) ratios in the precursors.

In/(In+Ga) in the precursors	Peak wavelength / nm	PL QY / %
0.10	517	12
0.20	548	36
0.40	570	34
0.50	579	18

(a) The samples were prepared under the same conditions as those in Fig. 4.

Table S5. Chemical compositions of Na-AIGS(1.0)(tu) QDs[(a)] prepared with different Na/(Ag+Na) ratios in the precursors.

Na/(Ag+Na) in the precursors	Fraction (%)					Charge balance (anion/cation)	Metal ratios	
	Na	Ag	In	Ga	S		Na/(Ag+Na)	In/(In+Ga)
0.30	4.4	1.3	0.7	63	30	0.31	0.77	0.011
0.50	3.7	1.0	1.0	63	31	0.31	0.79	0.016
0.70	6.0	12	7.7	32	42	0.61	0.33	0.19

(a) The samples were prepared under the same conditions as those in Fig. S5.

Table S6. PL peak wavelengths and quantum yields of Na-AIGS(1.0)(tu) QDs[(a)] prepared with different Na/(Ag+Na) ratios in precursors.

Na/(Ag+Na) in the precursors	Peak wavelength / nm	PL QY / %
0.30	548	36
0.50	550	26
0.70	575	0.6

(a) The samples were prepared under the same conditions as those in Fig. S5.

Table S7. Parameters to fit PL decay profiles of AIGS(1.5) QDs.

Samples	τ_1 (ns)	A_1 (%)	τ_2 (ns)	A_2 (%)	τ_3 (ns)	A_3 (%)	τ_{ave} (ns)	χ^2
AIGS(es)	5.7	55	37	36	175	8.5	99	1.20
Na-AIGS(es)	5.1	57	33	35	127	7.7	68	1.12
AIGS(tu)	40	75	175	25	---	---	120	1.16
Na-AIGS(tu)	34	64	109	36	---	---	83	1.17

(a) The samples were prepared under the same conditions as those in Fig. 1.
